# ‘Tied up in a legal mess’: The alcohol industry's use of litigation to oppose minimum alcohol pricing in Scotland

**DOI:** 10.3366/scot.2020.0304

**Published:** 2020-02

**Authors:** Benjamin Hawkins, Jim McCambridge

**Affiliations:** ^A^ Dr Benjamin Hawkins is Associate Professor at the London School of Hygiene and Tropical Medicine. His research focuses principally on the role of corporate actors, particularly in the alcohol and tobacco industries, on health policy debates at the national, European and global levels. In addition, he has published work on European integration, multi-level governance, international trade and political economy approaches to health policy.; ^B^ Professor Jim McCambridge holds the Chair in Addictive Behaviours & Public Health at the University of York. His research interests span alcohol, drugs and other addictions and research methodology. He is also Visiting Professor at Linkoping University in Sweden, and Conjoint Professor at the University of Newcastle in Australia. His alcohol policy work is primarily concerned with better understanding the ways in which corporate actors influence science and policy in order to strengthen public health policy making.

**Keywords:** alcohol policy, Minimum Unit Pricing, Scotland, legal challenge, alcohol industry

## Abstract

This article examines the alcohol industry's legal challenges to minimum unit pricing (MUP) in Scotland through the stages heuristic of the policy process. It builds on previous studies of alcohol pricing policy in Scotland and across the UK, and of the use of legal challenges by health harming industries to oppose health policy globally. Having failed to prevent MUP passing into law, industry actors sought to frustrate the implementation of the legislation via challenges in the Scottish, European and UK courts. However, the relevance of legal challenges is not limited to the post-legislative stage of the policy process but was foreshadowed in all earlier stages of the policy process. The *potential* for a legal challenge to MUP, and the alcohol industry's clearly articulated intention to pursue such action, was used by industry actors to seek to prevent the adoption of MUP in the agenda setting, policy formulation and legislative stages and created significant ‘regulatory chill’ in other areas of Scottish and UK alcohol policy. Litigation, and the prospect of it, was thus part of a coherent and integrated long-term strategy which adapted to changes in the political climate and to different stages in the policy process. While both the rhetoric and reality of litigation failed to prevent policy implementation, it succeeded in causing a delay of six years, imposing significant costs on the Scottish government and creating policy inertia in Scottish alcohol policy subsequently. Moreover, the inclusion of a ‘sunset clause’ in the legislation, requiring ongoing evaluation of the policy's effects, presents additional opportunities for the industry to reverse MUP. Thus, industry strategies to undermine MUP and delay further alcohol policy developments require ongoing attention by policy actors and scholars.

## Introduction

This article examines the role of litigation in alcohol industry strategy to oppose the introduction of minimum unit pricing (MUP) for alcohol in Scotland. It builds on previous analyses of MUP debates and corporate political strategies both in Scotland (Holden and Hawkins, 2012, McCambridge et al., 2013, Katikireddi et al, 2014a, 2014b, Katikireddi and Hilton, 2015, Katikireddi and McLean, 2012) and across the United Kingdom (UK) (Holden et al., 2012, Hawkins and Holden, 2013, 2014). As such, it is of relevance to scholars of alcohol policy, health policy, and public policy in Scotland and beyond. In addition, the article contributes also to the limited, yet expanding, literature on the role of legal challenges by corporate political actors globally to stymie the introduction of policies, which affect the business models and profits of health harming industries (McGrady, 2007, 2011, 2012 Voon and Mitchell, 2012a, Voon et al., 2014, Jarman, 2015, Hawkins and Holden, 2016, Hawkins et al., 2018, Holden and Hawkins, 2018a, 2018b).

The case of MUP is an example of a legal challenge brought under European Union (EU) single market law to policies introduced by a devolved administration within a member-states, in ways that reflect the specific competencies devolved to Scotland (i.e. health) and retained by the UK government (i.e. taxation) (see Holden and Hawkins, 2012, 2016). Consequently, the analysis below will be of relevance to those working on the topics of multi-level governance (MLG) and corporate political strategy in Scotland, the United Kingdom (UK), the EU and beyond (Hawkins et al., 2018, 2019).

Much of the literature on industry legal challenges focuses on the use of international trade and investment agreements to challenge domestic laws by the tobacco industry (McGrady, 2007, 2011, 2012, Voon and Mitchell, 2012b, Jarman, 2015, Hawkins and Holden, 2016, Hawkins et al., 2018, Holden and Hawkins, 2018b). While the tobacco industry has been the most litigious industry, reflecting its marginalisation from other forms of policy influence in many contexts, such agreements afford similar opportunities to industries in other sectors (Eberhardt and Olivet, 2012). This includes the alcohol industry's use of trade and investment agreements (Voon et al., 2014), and EU single market law to challenge MUP (Holden and Hawkins, 2016, 2018b).

The existing literature focuses almost exclusively on ‘live challenges;’ that is to say legal proceedings instigated against policies already adopted in an attempt to prevent or overturn their introduction. While these studies recognise the potential ‘chilling effect’ of litigation (Fooks and Gilmore, 2013, Côté, 2014, Hawkins and Holden, 2016) – whereby governments in other settings are deterred from adopting similar measures – such impacts (i.e. the absence of policy action) may be challenging to study. Moreover, little attention has been paid to ‘internal chilling effects’ or the effects of legal challenges on the wider policy developments within the policy context in which they are undertaken. The present study thus offers a valuable addition to our understanding of the relationship between legal challenges and policy making in the area of health, and public policy more generally, by documenting the role of legal challenges in shaping the policy process.

The argument developed below is that the potential for legal challenge under EU single market law was a vital component of the alcohol industry's political strategy throughout the Scottish MUP debates which had a key structuring effect on all stages of the policy process. The eventual initiation of legal proceedings by the Scotch Whisky Association (SWA) in the Court of Session in 2012 represented the adaptation and escalation of the alcohol industry's political strategy to prevent MUP coming into force after its adoption into law. However, the relevance of this litigation is not limited to the implementation stage of the policy process. It was a key industry influencing tool throughout the MUP debate.

To demonstrate this, the present study employs the ‘stages heuristic’ of the policy process as an analytic framework. This identifies sequential stages in policy development from the initial framing of social issues as ‘policy problems’ and their entry onto the policy agenda; through the development of potential solutions to these; to the enactment (or rejection), implementation and evaluation of interventions (Lasswell, 1956, Pressman and Wildavsky, 1973, Mazmanian and Sabatier, 1980). The analytical value of the stages heuristic for the present case lies in the way in which it highlights the importance of the post-legislative implementation and evaluation phases in the policy process. Even after the formal adoption of a policy – including the passage of new laws – policy actors such as the alcohol industry expend considerable efforts to block the implementation of such measures and to shape the ways in which they are evaluated and maintained. Such activities were evident in the case of MUP for alcohol in Scotland with significant consequences for Scottish policy making and for population health, which are yet to be fully examined in the scholarly literature.

Previous studies have found that the alcohol industry is capable of engaging in every stage of the policy process, through highly-sophisticated, multi-dimensional political strategies (McCambridge et al., 2014, 2018). This is in keeping with wider studies of the political strategies of the alcohol, tobacco and other health harming industries (McCambridge et al., 2018, Brandt, 2012, Kenworthy et al., 2016). Corporations in different sectors tailor their political strategies to the specificities of the policy spaces in which they are active and adapt these incrementally in response to changing circumstances, including developments at different stages of the policy process (Baron, 1995, Hillman et al., 2004, Shaffer and Hillman, 2000).

In this article, we identify how alcohol industry strategy evolved in response to developments in the alcohol policy process in Scotland and the implications of this for the subsequent development of alcohol policy in Scotland and elsewhere in the UK. The 2011 Scottish Elections, at which a majority Scottish National Party (SNP) government was elected on a manifesto commitment to introduce MUP, was a key juncture in both the development of alcohol pricing policy and in the evolution of industry strategy to oppose this. Prior to this, the industry had relied on political access and lobbying to stymie the development of MUP, but had continually adapted their approach to the changing political context in Scotland (Holden and Hawkins, 2012). The lack of well-developed industry connections with the 2007–2011 Scottish National Party (SNP) minority administration, and the political commitment that the government demonstrated to MUP, had led industry actors to shift their lobbying targets from ministers and officials in the agenda setting and policy development phases to opposition Members to the Scottish Parliament (MSPs) in the legitimation/legislative stage. This allowed them to successfully lobby for the removal of the MUP proposals from the 2010 Alcohol Etc. (Scotland) Act (Holden and Hawkins, 2012). After 2011 the parliamentary arithmetic meant it was no longer possible to stop MUP becoming law and the industry strategy evolved again.

Following the passage of the 2012 Alcohol (Minimum Pricing) (Scotland) Act, the industry shifted to towards more overtly confrontational strategies, most obviously the legal challenge they mounted in the Scottish, European and UK courts. This was designed to prevent, or at least delay, the implementation of MUP and to absorb governmental resources, thereby undermining its capacity for further policy action and shaping the entire Scottish alcohol and health policy space. However, while legal challenges were clearly intended to stymie the post-legislative introduction of MUP, we suggest that its significance to MUP, and Scottish alcohol policy more generally, is not limited to this implementation phase. Rather, the potential for judicial review of MUP cast a shadow over the entire policy process. The commencement of legal actions in the Court of Session can be seen, therefore, as the final phase in a co-ordinated, sequential and multi-faceted political strategy, which planned for, and adapted to, different eventualities and outcomes at each stage of the policy process.

## The ‘stages heuristic’ of the policy process

The stages heuristic of the policy represents a simplified, ‘ideal type’ model of the policy process, which underplays the complexity and ‘messiness’ of policy-making and the overlapping, non-linear movement between the stages of this process which are captured by more nuanced, multifarious theorisations of the policy process. Consequently, its application in the field of policy studies has been subject of some critique from those working within other frameworks (see Nakamura, 1987, Sabatier and Jenkins-Smith, 1993). These limitations notwithstanding, the stages heuristic remains a useful device for conceptualising the evolution of policy debates and the evolution of policy actors' political strategies at different points in the process. In this article, it provided the framework through which we conceptualised the impact of legal challenges at different stages of the MUP policy process policy and informed the analysis of the conceptualisation of the current article and the analysis of the interview data from which this article emerges.

Whilst the precise terminology employed to describe each stage in the policy process has evolved over time and varies between studies, proponents of the stages heuristic identify and examine seven principal stages of the policy-making process: agenda setting, policy formulation, legitimation (i.e. legislation and/or enactment), implementation, evaluation and policy maintenance. *Agenda setting* refers to the process through which social objects become identified as policy problems requiring a governmental response. The *policy formulation* phase involves identifying concrete responses to policy problems and gaining the political commitment to enact them.

The *legitimation* phase refers to the various means through which policies are enacted and through which consent is sought for their adoption. In democratic settings, this occurs most commonly through the passage of legislation in which popular consent is transmitted via elected representatives in parliament and assemblies. In the case of MUP, the legitimation phase focussed on the highly contested attempts to pass legislation on MUP through the Scottish Parliament between 2010 and 2012. For this reason, and to ensure analytical clarity, we refer to this stage as the legitimation/legislative phase in the present article. However, the wider process of legitimation can be seen to encompass the justifications and support for policies that feed into parliamentary deliberations and decision-making, including the identification and appraisal of supporting evidence for the scale of a policy problem and the effectiveness of the proposed intervention in the period from the 2008 MUP consultation onwards. This tends to be the phase of the policy process, which receives most attention from both scholars and the wider public. It offers concrete events, actions and processes (i.e. policy documents and Bills, speeches and votes in parliament) which receive media attention and offer themselves as objects of study. This, however, risks underplaying the crucial role of agenda setting and policy formulation in defining the terms and parameters of policy debates, which create path dependencies towards certain policy options while excluding others. For these reasons, policy framing has been identified as a vital component of the Scottish alcohol policy making process (Katikireddi et al, 2014a, Hawkins and Holden, 2013) as policy actors seek to influence the policy debates at the earliest possible juncture (Hawkins and Holden, 2014, Lukes, 1974).

The *implementation* stage of the policy process has received less attention from policy scholars and has a lower prominence in the public consciousness than the legitimation/legislative stage. This is perhaps due to the perception that, once a policy is enacted, the debate is over or the issue has been resolved. There is a widespread misconception that ‘implementation should be easy’ (Pressman and Wildavsky, 1973). In fact, many of the most arduous aspects of policy debates can relate to the specific details and mechanics of how a policy will be implemented. Well-resourced policy actors are able to employ various strategies to seek to prevent or delay the introduction of even democratically approved policy measures, particularly those characterised by technical complexities. There is little evidence on alcohol industry strategies in this stage (McCambridge et al., 2018). In the case of MUP, the implementation phase was dominated by an industry-led legal challenge to the policy in the Scottish, European and UK courts. *Policy evaluation* is also of vital importance to the success of a policy, and the Scottish Government has put in place mechanisms to evaluate the effectiveness of the policy and a ‘sunset clause’ (see below) which will be vital to the long-term *maintenance* of the policy.

It is the implementation and evaluation stages of the policy process, which form the basis of the analysis of the Scottish MUP debates below. Whilst implementation was significantly delayed, and evaluation remains ongoing, we document how industry actors sought, and continue to seek, to shape these phases of the policy process. The article emerges from a wider study of UK alcohol policy since 2010, focussing on MUP (in both England and Scotland) and wider policy developments across the UK in this period, including the public health responsibility deal (for England) and the 2016 revision of the Chief Medical Officers' drinking guidelines. The analysis below draws on 26 semi-structured interviews (see Brinkmann, 2013, Rubin and Rubin, 2012) conducted between February and October 2018 with policy actors (parliamentarians, civil servants, civil society actors and academics) in Edinburgh and London, which focussed on the range of topics and events examined in the study. Interviews sought to understand the timing and dynamics of policy debates, and the roles played by policy actors inside and outside of government (including but not limited to the alcohol industry) in determining the outcomes of alcohol policy making in this timeframe. Respondents were identified and recruited using purposive and snowball sampling following a stakeholder analysis (Varvasovszky and Brugha, 2000, Brugha and Varvasovszky, 2000). Interviews were audio-recorded, transcribed and analysed using thematic coding (see Braun and Clarke, 2006).

## The agenda setting, policy formulation and legislative stages: the rhetorical uses of the legal challenge

The period before 2011 covered the *agenda setting* and *policy formulation* stages of the Scottish MUP debate and the beginning of the legitimation/ legislative stage, which culminated in the 2007–2011 SNP minority government's unsuccessful attempt to pass MUP into law via the 2010 Alcohol Etc. (Scotland) Act. The legitimation/legislation stage ended, and the implementation stage began, in 2012 with the passage of MUP into law in the Alcohol (Minimum Pricing) (Scotland) Act 2012 under the SNP majority government. Whilst the legal challenge of the Scotch Whisky Association (SWA) to MUP was initiated only after the passage of MUP into law, the prospect of a potential court case cast a shadow over the entire policy process, supporting different aspects of industry strategy in different phases. It is important, therefore, to see the legal challenge both in instrumental terms and as having important rhetorical and symbolic dimensions. The rhetorical threat of legal challenge was designed to prevent the MUP legislation passing in the first instance but, should this strategy fail, the industry would be seeking to overturn, or at least delay, the implementation of the policy via actual litigation. The prospect of defending a legal challenge – and the implications this would have for the Scottish Government – was raised by industry actors during the policy formation and legitimation/legislative phases in an attempt to shape the policy debate and undermine the Scottish Government's commitment to introducing the policy. This created additional complications in the legislative process – for example, the need to seek additional legal opinions and guarantees before proceeding – which slowed its conclusion (see Hawkins and Holden, 2016). As one NGO respondent commented:

[The SWA] made it very clear from the start, that there would be a legal challenge, and it would be a difficult legal process. […] I think as soon as the issue was aired, that that was a key plank of their argument. And […] they kind of presented a position that they understood the international legal dimension, particularly the European legal dimension, in a way that the Scottish Government didn't. So, they presented that they were involved in trade discussions around the world all the time, and they were very experienced at it, and were doing it all the time […]. That was pretty much their angle right from the start, and it was the Scotch Whisky Association that we were hearing most from on that. […] But I think it was presented in a tone of, ‘you know, we've been involved in these very complicated negotiations that you know nothing about, take it from us.’

This account was supported by civil servants involved in the policy process who recalled industry actors raising the potential of legal challenges on the very day on which former Justice Secretary, Kenny MacAskill, set out the Government's intention to introduce MUP in March 2009.

As noted by previous studies, prior to 2010 opposition parties were the key conduit for the industry's efforts to prevent MUP (Holden and Hawkins, 2012). This included statements that the Scottish Government would face a legal challenge and provided grounds for them to oppose MUP. As one civil servant familiar with the MUP process commented:

[The industry] were getting at the opposition by saying, ‘well, this legislation is open to challenge.’ And the opposition parties, who had already sort of made up their mind to oppose it, were actually jumping on that as well. They were saying it's open to challenge so how can we support it. […] The threat of legal action was constant and consistent throughout the entire process really from day one.

The significant resources at the industry's disposal allowed them to commission legal opinions, which became important points of reference within the policy debate, and a pretext for contacting and engaging key policy actors, including the core policy team working on MUP and other actors across government, who may be affected by the legal implications of the policy. As the previously quoted civil servant continued:

[They were] commissioning opinions from various counsel, which was sort of backing up their position […] and writing in to ministers in sort of fairly general terms to say, we have counsel's opinion which says this is contrary to competition law because of X, Y and Z. And that was pretty constant throughout. When they weren't getting any traction […], they were then trying other bits of Government at fairly senior levels. So, for example the director responsible for enterprise and industry was getting letters from SWA saying, what these guys in Health and Justice are doing is contrary to European law. And there was various bits of Government, very senior officials, that were being picked off to try and get them to put pressure on us to say: ‘are you sure you know what you're doing?’ […] some fairly senior people saying: ‘you do know the sort of implications of what you guys are doing?’

## From legislation to implementation: litigating MUP

Following the election of the SNP majority government committed to introduction of MUP in 2011 there was also a realisation amongst industry actors and other stakeholders that the passage of the measures into law had become inevitable. As one NGO actor explained:

The political arithmetic changed thereafter […] the political debate was over, so the SNP, the Greens, the Liberal Democrats, and the Conservatives were all, you know, supportive of the policy, and wanted to see it come in.

This was echoed by a representative from the Scottish Government:

My reading of it is that there was an understanding by the opponents of MUP that because there was a majority government now – and because, you know, they've been elected with a very clear manifesto commitment to do MUP – that […] it was going to happen one way or another, so the shift in focus then is from […] lobbying around parliament with the 2010 legislation to then trying to find ways to prevent the implementation of the legislation after 2011 and principally through the legal challenge.

The Alcohol (Minimum Unit Pricing) (Scotland) Act received Royal Assent in June 2012. In July that year, the SWA launched a challenge to the legislation in the Court of Session in Edinburgh, on the basis that it infringed both UK (including the 1707 Act of Union) and European Union (EU) single market law (see Hawkins and Holden 2016). The Outer House of the Court of Session found in favour of the Scottish Government, but the SWA appealed that decision. The Inner House of the Course of Session (the Court of Appeal) referred the case to the Court of Justice of the European Union (CJEU) for a preliminary ruling on the relevant aspects of EU law in April 2014. The Opinion of the Advocate General preceded a formal judgement by the CJEU in December 2015, before the matter was passed back to the Scottish courts to rule on the case. The Inner House found in favour of the Scottish Government in July 2016, but the decision was again appealed by the SWA. The UK Supreme Court finally decided the case in favour of the Scottish Government in November 2017, exhausting all legal avenues open to the SWA. MUP was implemented in May 2018: six years after its passage into law, and seven years after the SNP majority government was elected on a manifesto commitment to introduce the policy. However, the rationale for pursuing legal challenges so exhaustively was not limited to the desire to block the implementation of the policy but served a wider purpose within industry actors' overall political strategies.

## Shaping perceptions of the legal case

At various stages of the legal process, particularly while the case was before the European court, industry actors sought to shape perceptions of the MUP case and its likely outcome as different judgements were delivered. As one NGO respondent commented: ‘They would constantly put out PR saying that they had won and that the case had been defeated, when in fact it hadn't been.’ There was an attritional character to the strategy, apparent in another NGO actor's account, which suggested their objective was ‘to get the government to give up, to get the government to accept defeat’ in their defence of the case.

NGOs responded by offering their own unequivocal interpretations of the judgements, arguing the courts supported the Scottish Government's positions, despite some internal doubts about the potential outcome of the case. As one NGO respondent commented:

I guess my attitude on this was just not to ever admit any doubt about it. […] And I just kept on constantly, constantly, constantly saying that and trying to get other people into that space as well. And I think that was the right thing to do.

Engaging public opinion, and shaping perceptions about the feasibility of MUP, were thus a key component of the policy process as policy actors on both sides sought to undermine or buttress the political commitment of the government in light of this.

## Draining resources

The lengthy series of legal challenges represented a significant drain on the Scottish Government's resources. Asked about this, a civil servant familiar with the government's defence of the action commented:

Very, very significantly, both directly and indirectly […]. [There was] a division at the time with 70 odd people, one of the biggest divisions in the government, and which dealt with things like vaccination policy, [… plain packaging of cigarettes] and other aspects of booze and obesity and all of that stuff and running multiple bits of legislation and sometimes multiple court cases. But this was the most important, this was the thing that [the division] needed to invest time in most and there were several people both within [that division] and in wider teams in the legal directorate and in the statistic analytical services team who were, for very long periods of time, doing little else apart from this.

[…] I think it may even have been above 10,000 pages of material and reports are in front of it. […] Somebody needed to have read all of that and to keep up with it and to be thinking about the best way, the best approach to take in court and to be reading the other side's material and thinking also about […] where it was going to go to next because it was perfectly plain, particularly in the earlier court hearings, it was perfectly plain that whoever won the first round that was not the end of it […] where is it going to next and is there anything that we should be doing now to prepare for that.

Much of the court case turned on the effectiveness of MUP in achieving the stated goals of the Scottish Government and the inability of other possible measures to achieve the same outcomes through less trade restrictive measures (see Hawkins and Holden, 2016, Holden and Hawkins, 2018a). The SWA submitted significant volumes of information and evidence to the court, including critiques of the Scottish Government's evidence in support of MUP. This placed significant time pressures on both the Scottish Government, and the researchers providing this evidence, to respond to such industry submissions.

## Block, amend, delay

Respondents in this study were unanimous in their belief that the primary purpose of the legal challenges was to prevent the introduction of MUP. Failing this, however, their secondary objective was to delay its introduction as long as possible. Whilst they failed to prevent the introduction of MUP, they were able to delay its implementation significantly.

The duration of the legal case and the extent to which the SWA was prepared to exhaust all possible avenues available to them surprised some government actors. As one civil servant commented:

[…] it was appeal after appeal, and challenge after challenge, right up to the Supreme Court. I never really thought they would go that far. […] It just surprised me how much resource they put into trying to scupper this.

This was reflected by NGO respondents, who felt the decision to pursue the legal challenge to the bitter end was a strategic mistake on the part of the SWA. In so doing, they misjudged both the determination of the government to act on price and the mood of the population who were convinced of the need for decisive action on alcohol and were prepared to accept price-based measures. According to a former Minister:

We always had fall-back positions. Plus, we had big public support saying, ‘well, that's wrong.’ […] They weren't wired into communities, and they weren't picking that up. And I think that just comes back to a general arrogance. You know, we're the Scotch Whisky Association, you know, we do what's good for you, you should know that. You know, you [the government] do what we tell you.

The doggedness of their actions was seen some respondents as reflecting a determination to avoid the regulatory fate which had befallen the tobacco industry in previous decades. Whilst the SWA failed to prevent the introduction of MUP, they did succeed in delaying its introduction significantly in Scotland. This, in turn, prevented or slowed its adoption in England, Wales and elsewhere (Hawkins and McCambridge, 2019). As a former civil servant commented, this delay represented a significant achievement for the industry:

[…] if we see their objective as not bringing it in at all they failed. But, actually, in the meantime they delayed it. They've tied it all up in knots. They've created a lot of doubt about it.

Delays of this kind are useful to industry actors and mirror the ‘block, amend, delay’ tactics used by the tobacco industry in opposing the 2014 EU tobacco products directive (Peeters et al., 2015). The ability to delay legislation allows other events to intervene. There may be a change of government and political direction, a shift in public opinion, or a crisis to which government time and resources may be diverted. Even measures that seem inevitable may never come to pass. In the interim, businesses can continue to exploit the current arrangements and can begin to adapt their business models and market strategies in response to proposed policies. Moreover, they can seek to undermine or weaken measures in ways amenable to their commercial interests. Finally, delay may render aspects of the policy out of date and thus less impactful. For example, respondents identified that the proposed 50p rate of MUP proposed in 2012 would in fact be a lower price in real terms by 2018 because of inflation over the intervening six years.

## Policy inertia

Whilst welcoming the introduction of MUP and recognising the scale of the task faced by the Scottish Government to bring this into effect, some respondents felt that this battle had been won at the expense of other areas of alcohol policy, in which progress has subsequently stalled. There was a concern amongst alcohol advocates that, following the passage of such a high-profile policy as MUP, the Scottish Government's position was that alcohol had been dealt with and no further action was needed. There was also a sense, respondents felt, that alcohol had had its fair share of time and resources and that it was now the turn of related issues such as obesity. This was related to perceptions of resource exhaustion (see above) and reluctance to take on other controversial issues, which may also face significant industry opposition. As one NGO respondent commented:

The implementation of minimum pricing, and seeing it through to the court case, and the secondary legislation that's been required, all of that has been led by the Health team […] [T]hat has completely occupied that team. So, you know, we should have had [a refreshed alcohol strategy] more than two years ago. And it still hasn't issued. So, I think, you know, it has just completely soaked up the effort of that team, over that time, and before.

Some respondents believed that alcohol policy developments have been undermined by cuts in the numbers of civil servants and the different policy priorities of incoming ministers who were less interested in alcohol. Yet the MUP experience clearly also played a part in this. The same respondent continued:

I think, you know, overriding all of that is a kind of sense of regulatory chill of, ‘well, we've been tied up in this legal mess around MUP for a number of years, […] what can we do that isn't gonna (sic) get us in court again?’ […] So, we're in a bizarre situation in relation to marketing, where there's a much stronger evidence base around alcohol marketing, and the harms to children, than there is around junk food marketing, and harm to children. And yet, as things currently stand, there is a potential will to legislate on that, but not on alcohol.

This was supported by another NGO actor:

I mean, the problem we've got here though now is, I think that the government's gone cool on doing anything else that's going to tie them up in court […] In alcohol specifically. I think they've moved on to other issues. Obesity is far more of a priority and drugs is a priority, because we've got highest rates of drug deaths that we've ever had.

Given the wider effects that the MUP debates have had on Scottish alcohol policy and the political will to take on other issues, the industry's efforts to influence alcohol policy were not completely unsuccessful. The significant resources expended in opposing MUP failed to prevent its introduction but succeeded in delaying wider progress in alcohol policy, including the development of a new Scottish alcohol strategy (eventually published in November 2018), and slowed the diffusion of MUP to other parts of the UK (Hawkins and McCambridge, 2019). Perhaps most importantly, it sent a signal to the Scottish Government and policy makers elsewhere that the industry would fight policies regarded as detrimental to their interests to the bitter end, with significant political and material costs for any government.

## Evaluation

Evaluation is a vital stage in the policy process. Understanding the effectiveness of adopted measures is essential to support good governance and evidence informed policy. This, in turn, impacts on the maintenance of the policy and its acceptance amongst key stakeholders and the wider public. From an industry perspective, the evaluation stage offers opportunities to question and potentially undermine the effectiveness of this policy, to influence public support and to press for its repeal. The evaluation of MUP in Scotland was delegated to the Monitoring and Evaluating Scotland's Alcohol Strategy (MESAS) group within NHS Scotland (NHS Scotland, 2019).

In the case of MUP, the importance of the evaluation process was heightened by the insertion of a ‘sunset clause’ in the Alcohol (Minimum Unit Pricing) (Scotland) Act. This required the policy to be renewed in law after a specific implementation period, via a vote in the Scottish Parliament. In part, this was included in an attempt to dispel criticisms of, and reduce controversy surrounding, such a novel policy intervention. Respondents disagreed about whether this was useful in securing the passage of the legislation under the SNP majority government. However, the inclusion of the clause in the legislation proved important during the subsequent legal challenge. As a former Scottish Government actor commented:

It proved probably unnecessary in the Scottish Parliamentary procedures, but it became I think invaluable, […] later on in the process where what we were … in essence what we were saying was we think this will work.

This view was supported by an NGO representative, who saw the clause as vital in the context of the legal challenge:

I think it was pretty clear from the Supreme Court judgement, and also from the Court of Session hearings in Scotland, that the recognition that this measure was experimental, was one that I think the courts thought that was a good thing to have been acknowledged […] that the sunset clause and evaluation was an important part of them finding the measure to be, you know, having been appropriately considered by Parliament.

Industry actors sought involvement in the MESAS evaluation process, a request to which the Scottish Government acceded with the inclusion of industry representatives on one of the Economic Impact and Price Evidence Advisory Groups; one of five advisory bodies overseeing the evaluation (NHS Scotland, 2019). As one NGO respondent suggested:

So, that was a trade-off, when the Supreme Court Judgement was handed down, I believe. The Scotch Whisky Association met with Nicola Surgeon, I believe, and demanded that they were involved in the evaluation, that they'd stop their public opposition to the policy. And, they do, they sit on the Advisory Group to the evaluations.

Notwithstanding conflicts of interest and preceding events, industry participation in the evaluation process would permit ongoing access to policy-makers, and potential to influence decision-making.

NGO respondents questioned the industry's commitment to thorough, independent evaluation of the policy given their reluctance to share data; something that is at odds with the unique contribution that they claim to be able to make to policy development:

[It's] a frustration to me that we haven't had more of that contribution of the industry's knowledge of sales patterns, and influences on sales, as part of the process […] I think if they're talking about being part of the partnership, and understanding alcohol, consumption of alcohol, and harm in Scotland, that's part of their contribution. If they, you know, cut the price of a bottle of vodka in a supermarket, what impact does that have on sales? Because they have a higher level of knowledge and expertise in that. And none of that is now shared in the public interest.

Other respondents, meanwhile, were aware that producer companies and other industry bodies had recruited companies to undertake surveys on MUP on their behalf, in an attempt to shape perceptions of the policy's effectiveness outside the formal evaluation process. This approach of funding alternative studies in order to create parallel literatures on key policy issues mirrors alcohol and tobacco industry strategies previously documented (Hawkins and McCambridge, 2014, McCambridge et al., 2013, Jernigan, 2012, Stenius and Babor, 2010, Caetano, 2008, Brandt, 2012, Hurt et al., 2009).

## Conclusion

The use of legal challenges to unfavourable policies is in keeping with both past studies of alcohol industry political strategies and those of the global tobacco industry (Hawkins and Holden, 2016, Holden and Hawkins, 2018a, Jarman, 2015). Legal challenges are designed in the first instance to prevent the implementation of such policies. However, the importance of litigation to industry strategies, and its effect on policy, should not be seen in such narrow, transactional terms. Even where legal challenges fail to prevent a policy coming into force, they offer considerable strategic advantages to industry actors. They delay implementation, which allows the industry to continue profitable activities in the interim and buys time to develop alternative strategies to respond to the changing political context. Legal cases may also serve to undermine the credibility of policies in the eyes of key political actors and the wider public, while creating additional pressure to abandon the contested measures. In addition, legal challenges impose significant material, as well as political, costs on policy makers. Defending policies against legal actions places considerable burdens on government time and resources and undermines efforts to tackle other public policy issues. This is particularly problematic for policy makers in smaller nations, and in the context of decreasing governmental resources since the 2008 global financial crisis and subsequent austerity programmes.

Perhaps of even greater value to the industry is the extent to which the mere threat of legal challenges can significantly influence the development of policy debates and may deter governments from bringing forward unfavoured measures. While it is extremely difficult to capture non-action of this kind in response to legal threats or other stimuli, the current article gets closer to this than previous studies by using the responses of key actors in the policy process. We document how the potential of legal action was used by the industry and how it factored into governmental decision making in the MUP process and subsequent alcohol policy developments.

The Scottish MUP case appears to have had a clear structuring effect on how the Scottish Government handled both the development of MUP and the subsequent policy agenda, delaying for instance the launch of its updated alcohol policy strategy. In addition, the MUP case appeared to have a ‘chilling effect’ on governments elsewhere in the UK considering similar measures. The legal challenge to the Scottish Government's MUP policy was instrumental in the decision of the Westminster government to delay implementing MUP in England in 2013, with no subsequent plans for its introduction announced to date (Hawkins and McCambridge, 2019).

The significant financial and non-financial costs imposed by legal challenges mean that there are strong incentives for policy makers to avoid them wherever possible. Studies of tobacco industry litigation have found that the threat of legal action can have a similar ‘chilling effect’ on governments (Hawkins and Holden, 2016, Côté, 2014, Fooks and Gilmore, 2013), preventing the adoption of evidence-based but industry-opposed measures such as standardised tobacco packaging. This means the mere potential to mount legal challenges is a highly valuable tool for corporate political actors to have at their disposal; one which is relevant not just at the implementation stage when litigation occurs, but at all stages of the policy process. This article thus builds on previous studies by demonstrating how legal challenges form part of a comprehensive corporate political strategy that adapts to all stages of the policy process from the very earliest, pre-legislative stages onwards.

Alcohol industry involvement in the process of evaluating MUP is potentially problematic and requires further attention by policy makers, public health advocates and scholars. Influencing strategies are often subtle, indirect and hard to detect and require vigilance on the part of all actors involved in the process. This is especially important given the inclusion of a sunset clause in the legislation, which requires the policy to be reapproved by the Scottish Parliament after 5 years. The institutionalization of MUP has been prevented and political contestation over the policy has been kept alive by the sunset clause, and the outcome of the evaluation will be integral to determining the future of the policy in the context of wider political developments in Scotland. Since the findings of evaluations of this kind are often not clear-cut, the long-term maintenance of the policy is far from assured, and industry strategies are likely to evolve again as this process develops. This requires vigilance by policy makers and public health actors and offers additional opportunities for study for health policy scholars.

Finally, this article, in highlighting the integrated and adaptive nature of industry strategy, underlines the importance for policy researchers of focussing on all stages of the policy process from agenda setting to evaluation and beyond. It demonstrates that policy processes, especially in controversial issues involving powerful vested interests, are never definitively resolved and require ongoing engagement from policy actors to maintain even effective and well-designed policy regimes in the face of concerted opposition.

## References

[b1] Baron D. P. (1995). Integrated strategy: Market and nonmarket components. *California Management Review*, 37, 47–65.

[b2] Brandt A. M. (2012). Inventing conflicts of interest: a history of tobacco industry tactics. *American Journal of Public Health*, 102, 63–71. 22095331 10.2105/AJPH.2011.300292 PMC3490543

[b3] Braun V. & Clarke V. (2006). Using thematic analysis in psychology. *Qualitative Research in Psychology*, 3, 77–101.

[b4] Brinkmann S. (2013). *Qualitative Interviewing*, Oxford University Press.

[b5] Brugha R. & Varvasovszky Z. (2000). Stakeholder analysis: a review. *Health Policy and Planning*, 15, 239–246. 11012397 10.1093/heapol/15.3.239

[b6] Caetano R. (2008). About smoke and mirrors: the alcohol industry and the promotion of science. *Addiction (Abingdon, England)*, 103, 175–8. 10.1111/j.1360-0443.2007.02104.x 18199295

[b7] Côté C. (2014). *A chilling effect? The impact of international investment agreements on national regulatory autonomy in the areas of health, safety and the environment*. The London School of Economics and Political Science (LSE).

[b8] Eberhardt P. & Olivet C. (2012). *Profiting from injustice: How law firms, arbitrators and financiers are fuelling an investment arbitration boom* [Online] Available: http://corporateeurope.org/sites/default/files/publications/profiting-from-injustice.pdf [Accessed 07 January 2020].

[b9] Fooks G. & Gilmore A. B. (2014). International trade law, plain packaging and tobacco industry political activity: the Trans-Pacific Partnership. *Tobacco Control*, 23 (1); e1. 10.1136/tobaccocontrol-2012-050869 PMC3888632 23788606

[b10] Hawkins B. & Holden C. (2013). Framing the alcohol policy debate : industry actors and the regulation of the UK beverage alcohol market. *Critical Policy Studies*, 7, 53–71.

[b11] Hawkins B. & Holden C. (2014). ‘Water dripping on stone’? Industry lobbying and UK alcohol policy. *Policy and Politics*, 42, 55–70.

[b12] Hawkins B. & Holden C. (2016). A corporate veto on health policy? Global constitutionalism and investor–state dispute settlement. *Journal of Health Politics, Policy and Law*, 41, 969–995. 10.1215/03616878-3632203 PMC5040601 27256810

[b13] Hawkins B., Holden C. & Mackinder S. (2018). A multi-level, multi-jurisdictional strategy: Transnational tobacco companies’ attempts to obstruct tobacco packaging restrictions. *Global Public Health*, 14 (4): 570–583. 29521160 10.1080/17441692.2018.1446997 PMC6129412

[b14] Hawkins B., Holden C. & Mackinder S. (2019). *The Battle for Standardized Packaging in Europe*, Basingstoke, Palgarave Pivot.

[b15] Hawkins B. & McCambridge J. (2014). Industry actors, think tanks, and alcohol policy in the United Kingdom. *American Journal of Public Health*, 104, 1363–1369. 24922137 10.2105/AJPH.2013.301858 PMC4103247

[b15a] Hawkins B. and McCambridge J. (2019). Policy windows and multiple streams: an analysis of alcohol pricing policy in England. *Policy and Politics* [Online]. Available at: 10.1332/030557319X15724461566370.

[b16] Hillman A., Keim G., Schuler D. & Keim G. D. (2004). Corporate political activity: A review and research agenda. *Journal of Management*, 30, 837–857.

[b17] Holden C. & Hawkins B. (2012). ‘Whisky gloss’: The alcohol industry, devolution and policy communities in Scotland. *Public Policy and Administration*, 28, 253–273.

[b18] Holden C. & Hawkins B. (2018a). Law, market building and public health in the European Union. *Global Social Policy*, 18**,** 45–61.

[b19] Holden C. & Hawkins B. (2016). Health policy, corporate influence and multi-level governance: The case of alcohol policy in the European Union. In: Kenworthy N., Mackenzie R. & Lee K. () *Case Studies on Corporations and Global Health Governance: Impacts, Influence and Accountability*. London: Rowman and Littlefield.

[b20] Holden C. & Hawkins B. (2018b). Trade and investment agreements and the global politics of health. In: McInnes C. L., K; Youde J, () *Oxford Handbook of Global Health Politics*. Oxford: Oxford University Press.

[b21] Holden C., Hawkins B. & McCambridge J. (2012). Cleavages and co-operation in the UK alcohol industry: a qualitative study. *BMC Public Health*, 12, 483–483. 22734630 10.1186/1471-2458-12-483 PMC3406968

[b22] Hurt R. D., Ebbert J. O., Muggli M. E., Lockhart N. J. & Robertson C. R. (2009). Open doorway to truth: legacy of the Minnesota tobacco trial. *Mayo Clinic Proceedings*, 84, 446–56. 19411441 10.1016/S0025-6196(11)60563-6 PMC2676127

[b23] Jarman H. (2015). *The Politics of Trade and Tobacco Control*, Basingstoke, Palgrave Macmillan.

[b24] Jernigan D. H. (2012). Global alcohol producers, science, and policy: the case of the International Center for Alcohol Policies. *American Journal of Public Health*, 102, 80–9. 22095330 10.2105/AJPH.2011.300269 PMC3490544

[b25] Katikireddi et al (2014a). Changing policy framing as a deliberate strategy for public health advocacy: a qualitative policy case study of minimum unit pricing of alcohol. *The Milbank Quarterly*, 92, 250–283. 24890247 10.1111/1468-0009.12057 PMC4089371

[b26] Katikireddi et al (2014b). Understanding the development of minimum unit pricing of alcohol in Scotland: a qualitative study of the policy process. *PLoS One*, 9, e91185. 24670519 10.1371/journal.pone.0091185 PMC3966757

[b27] Katikireddi S. & Hilton S. (2015). How did policy actors use mass media to influence the Scottish alcohol minimum unit pricing debate? Comparative analysis of newspapers, evidence submissions and interviews. *Drugs: Education, Prevention and Policy*, 22, 125–134. 10.3109/09687637.2014.977228 PMC4438355 26045639

[b28] Katikireddi S. V. & Mclean J. A. (2012). Introducing a minimum unit price for alcohol in Scotland: considerations under European Law and the implications for European public health. *European Journal of Public Health*, 22, 457–8. 22829492 10.1093/eurpub/cks091

[b29] Kenworthy N., Mackenzie R. & Lee K. (2016). *Case Studies on Corporations and Global Health Governance: Impacts, Influence and Accountability*. London: Rowman and Littlefield.

[b30] LasswelL H. D. (1956). *The Decision Process: Seven Categories of Functional Analysis*. College Park. Maryland: Bureau of Government Research, University of Maryland.

[b31] Lukes S. (1974). *Power: A Radical View*. London and New York: Macmillan.

[b32] Mazmanian D. A. & Sabatier P. A. (1980). A multivariate model of public policy-making. *American Journal of Political Science*, 439–468.

[b33] McCambridge J., Hawkins B. & Holden C. (2013). Industry use of evidence to influence alcohol policy: a case study of submissions to the 2008 Scottish government consultation. *PLoS Medicine*, 10, e1001431–e1001431. 10.1371/journal.pmed.1001431 PMC3635861 23630458

[b34] McCambridge J., Hawkins B. & Holden C. (2014). The challenge corporate lobbying poses to reducing society's alcohol problems: insights from UK evidence on minimum unit pricing. *Addiction*, 109 (2), 199–205. Available: http://onlinelibrary.wiley.com/doi/10.1111/add.12380/full. 24261642 10.1111/add.12380 PMC3992904

[b35] McCambridge J., Mialon M. & Hawkins B. (2018). Alcohol industry involvement in policymaking: a systematic review. *Addiction*, 113, 1571–1584. 10.1111/add.14216 PMC6100095 29542202

[b36] McGrady B. (2007). Trade liberalisation and tobacco control: moving from a policy of exclusion towards a more comprehensive policy. *Tobacco Control*, 16, 280–3. 17652245 10.1136/tc.2006.019141 PMC2598543

[b37] McGrady B. (2011). *Trade and Public Health: the WTO, Tobacco, Alcohol, and Diet*. Cambridge, Cambridge University Press.

[b38] McGrady B. (2012). Implications of ongoing trade and investment disputes concerning tobacco: Philip Morris v. Uruguay. In: Voon T., Mitchell A., Liberman J. & Glyn Ayres G. (eds.) *Public Health and Plain Packaging of Cigarettes: Legal Issues* Cheltenham: Edward Elgar.

[b39] Nakamura R. T. (1987). The textbook policy process and implementation research. *Review of Policy Research*, 7, 142–154.

[b40] NHS Scotland. (2019). *Overview of Evaluation of MUP* [Online]. Available: http://www.healthscotland.scot/health-topics/alcohol/evaluation-of-minimum-unit-pricing/governance-of-mup-evaluation [Accessed 5 April 2019].

[b41] Peeters S., Costa H., Stuckler D., McKee M. & Gilmore A. B. (2015). The revision of the 2014 European tobacco products directive: an analysis of the tobacco industry's attempts to ‘break the health silo’. *Tobacco Control*, 25, 108–117. 25713313 10.1136/tobaccocontrol-2014-051919 PMC4669229

[b42] Pressman J. & Wildavsky A. (1973). *Implementation*. Berkeley, California. University of California Press.

[b43] Rubin H. J. & Rubin I. S. (2012). *Qualitative Interviewing: The Art of Hearing Data*, London, Sage.

[b44] Sabatier P. A. & Jenkins-Smith H. C. (1993). *Policy Change and Learning: An Advocacy Coalition Approach*, Boulder. CO: Westview.

[b45] Shaffer B. & Hillman A. M. Y. J. (2000). The development of business – government strategies by diversified firms. *Strategic Management Journal*, 190, 175–190.

[b46] Stenius K. & Babor T. F. (2010). The alcohol industry and public interest science. *Addiction*, 105, 191–8. 19804465 10.1111/j.1360-0443.2009.02688.x

[b47] Varvasovszky Z. & Brugha R. (2000). A stakeholder analysis. *Health Policy and Planning*, 15, 338–345. 11012410 10.1093/heapol/15.3.338

[b48] Voon T., Mitchell A., Islands C. C. C., Mitchell H. C. C. A. & Liberman J. (2014). *Regulating Tobacco, Alcohol and Unhealthy Foods: The Legal Issues*. Abingdon: Routledge.

[b49] Voon T. S. & Mitchell A. D. (2012a). Implications of international investment law for plain tobacco packaging: lessons from the Hong Kong–Australia BIT. In: Voon T., Mitchell A., Liberman J. & Glyn Ayres G. (eds.), *Public Health and Plain Packaging of Cigarettes: Legal Issues*. Cheltenham: Edward Elgar.

[b50] Voon T. S. & Mitchell A. D. (2012b). Implications of WTO law for plain packaging of tobacco products. In: Voon T., Mitchell A., Liberman J. & Glyn Ayres G. (eds.), *Public Health and Plain Packaging of Cigarettes: Legal Issues*. Cheltenham: Edward Elgar.

